# Acute effects of fine particulate air pollution on ST segment height: A longitudinal study

**DOI:** 10.1186/1476-069X-9-68

**Published:** 2010-11-08

**Authors:** Fan He, Michele L Shaffer, Sol Rodriguez-Colon, Edward O Bixler, Alexandros N Vgontzas, Ronald W Williams, Rongling Wu, Wayne E Cascio, Duanping Liao

**Affiliations:** 1 Department of Public Health Sciences, Penn State University College of Medicine, A210, 600 Centerview Dr. Suite 2200, Hershey, PA, 17033, USA; 2 Sleep Research & Treatment Center, Department of Psychiatry, Penn State University College of Medicine, 500 University Dr., Hershey, PA, 17033, USA; 3 Exposure Measurements and Analysis Branch, Human Exposure and Atmospheric Sciences Division, U.S. EPA, MD-E205-04, Research Triangle Park, NC, 27711, USA; 4 Department of Cardiovascular Sciences, Brody School of Medicine, and the East Carolina Heart Institute and East Carolina University, Ste C, 2315 Executive Cir, Greenville, NC, 27834, USA

## Abstract

**Background:**

The mechanisms for the relationship between particulate air pollution and cardiac disease are not fully understood. Air pollution-induced myocardial ischemia is one of the potentially important mechanisms.

**Methods:**

We investigate the acute effects and the time course of fine particulate pollution (PM_2.5_) on myocardium ischemic injury as assessed by ST-segment height in a community-based sample of 106 healthy non-smokers. Twenty-four hour beat-to-beat electrocardiogram (ECG) data were obtained using a high resolution 12-lead Holter ECG system. After visually identifying and removing all the artifacts and arrhythmic beats, we calculated beat-to-beat ST-height from ten leads (inferior leads II, III, and aVF; anterior leads V3 and V4; septal leads V1 and V2; lateral leads I, V5, and V6,). Individual-level 24-hour real-time PM_2.5 _concentration was obtained by a continuous personal PM_2.5 _monitor. We then calculated, on a 30-minute basis, the corresponding time-of-the-day specific average exposure to PM_2.5 _for each participant. Distributed lag models under a linear mixed-effects models framework were used to assess the regression coefficients between 30-minute PM_2.5 _and ST-height measures from each lead; i.e., one lag indicates a 30-minute separation between the exposure and outcome.

**Results:**

The mean (SD) age was 56 (7.6) years, with 41% male and 74% white. The mean (SD) PM_2.5 _exposure was 14 (22) μg/m^3^. All inferior leads (II, III, and aVF) and two out of three lateral leads (I and V6), showed a significant association between higher PM_2.5 _levels and higher ST-height. Most of the adverse effects occurred within two hours after PM_2.5 _exposure. The multivariable adjusted regression coefficients β (95% CI) of the cumulative effect due to a 10 μg/m^3 ^increase in Lag 0-4 PM_2.5 _on ST-I, II, III, aVF and ST-V6 were 0.29 (0.01-0.56) μV, 0.79 (0.20-1.39) μV, 0.52 (0.01-1.05) μV, 0.65 (0.11-1.19) μV, and 0.58 (0.07-1.09) μV, respectively, with all p < 0.05.

**Conclusions:**

Increased PM_2.5 _concentration is associated with immediate increase in ST-segment height in inferior and lateral leads, generally within two hours. Such an acute effect of PM_2.5 _may contribute to increased potential for regional myocardial ischemic injury among healthy individuals.

## Background

Numerous studies have consistently found a significant association between exposures to fine particulate matter, defined as fine particles with aerodynamic diameter less or equal to 2.5 micrometers (PM_2.5_), and the risk of clinical cardiovascular disease (CVD) [1-4]. The mechanisms responsible for such an association have been the focus of recent environmental health studies. Several studies [5-10] have suggested that electrocardiographic (ECG) morphologies indicative of myocardial ischemic damages are significantly associated with short-term increases in PM levels, thus air pollution-induced myocardial ischemia is one of the potentially important mechanisms. No studies have been published documenting the exact time course of action from PM_2.5 _exposure to myocardial ischemia. On the other hand, ST-height changes, including both ST depression and elevation, generally have been accepted as indicators of myocardial ischemic injury [11,12]. We therefore designed this study to systematically investigate the effects and the time course of individual-level exposures to PM_2.5 _on the ST-height, an ECG marker of myocardial ischemia, in a community-based sample of middle-aged adults.

## Methods

### Study Design

For this report, we used the data collected for the Air Pollution and Cardiac Risk and its Time Course (APACR) study, which we designed to investigate the mechanisms and the time course of the adverse effects of PM_2.5 _on cardiac electrophysiology, blood coagulation, and systemic inflammation. Recruitment methods and examination procedures for the APACR study have been published elsewhere [13,14]. Briefly, all study participants were recruited from communities in central Pennsylvania, primarily from the Harrisburg metropolitan area. All participants gave written informed consent prior to their participation in the study. The inclusion criteria for the study included nonsmoking adults ≥ 45 years old who had not been diagnosed with severe cardiac problems (defined as diagnosed valvular heart disease, congenital heart disease, acute myocardial infarction or stroke within six months, or congestive heart failure). Community recruitment specialists from the Pennsylvania State University General Clinical Research Center (GCRC), which is funded by the National Institutes of Health, at the Penn State College of Medicine, and the GCRC-organized community outreach activities, supported the recruitment of the participants. The GCRC maintains a list of individuals who live in central Pennsylvania communities for various health-related studies. Approximately 75% of the individuals who were contacted and who met our inclusion criteria were enrolled in the APACR study. Our targeted sample size was 100 individuals, and we enrolled and examined 106 individuals for the APACR study. The examination of two participants per week was conducted from November 2007 to June 2009 for the entire examination period except for major holidays.

Study participants were examined in the GCRC in the morning between 8:00 AM and 10:00 AM on Day-1. All participants fasted for at least eight hours before the clinical examination. After completing a health history questionnaire, a trained research nurse measured seated blood pressure three times, height, and weight, and drew 50 mL of blood for biomarker assays according to the blood sample preparation protocols. A trained investigator connected the PM_2.5 _and Holter ECG recorders. Participants were given an hourly activity log to record special events that occurred over the next 24 hours, including outdoor activities, exposure to traffic on the street, traveling in an automobile, and any physical activities. The entire clinical examination session lasted about one hour. Participants were then released to proceed with their usual daily routines. The next morning, the participants returned to the GCRC to remove the PM_2.5 _and Holter monitors, deliver the completed activity log, have another 50 mL of blood drawn, and a urine sample collected. Then, an exercise echocardiography was performed on each participant according to a standardized protocol to measure the participant's ventricular function and structure. The entire Day-2 session lasted for about one hour and 45 minutes. Penn State University College of Medicine Institutional Review Board approved the study protocol.

### Personal PM_2.5 _Exposures

The APACR study used a personal PM_2.5 _DataRam (pDR, model 1200, Thermo Scientific, Boston, MA) for real-time 24-hour personal PM_2.5 _exposure assessment. Details of the exposure assessment [13,14] and of the instrument's performance have been reported elsewhere [15-18]. Real-time PM_2.5 _concentrations were initially recorded at one-minute intervals. For each participant, we calculated the 30-minute segment-specific averages, based on the whole and half hours, as our PM_2.5 _exposure variable in the APACR study. Therefore, the PM_2.5 _exposure variables were treated as repeated measures, and each individual contributed 48 exposure data points.

### Continuous Ambulatory ECG and ST-Height Variables

A high-fidelity (sampling frequency 1,000 Hz) 12-lead HScribe Holter System (Mortara Instrument, Inc., Milwaukee, WI) was used to collect the 24-hour Holter beat-to-beat ECG data. The high-fidelity ECG significantly increases the resolution and enhances the accuracy of various wave form measurements. The details of the Holter ECG data collection and reading have been published [13,14]. Relevant to this report, the Holter ECG data are scanned to a designated computer for offline processing by an experienced investigator using modified Holter System software and the SuperECG software (also developed by Mortara Instrument, Inc.). The main objectives of the offline processing were to verify the Holter-identified ECG waves, to identify and label additional electronic artifacts and arrhythmic beats in the ECG recording, and to perform beat-to-beat ECG analysis to calculate various ECG waveform parameters, including ST segment height. The calculated ST-height included beat-to-beat ST-height from ten leads (inferior leads II, III, and aVF; lateral leads I, V5, and V6; septal leads V1 and V2; and anterior leads V3 and V4), defined as the distance between the J point, and 60 ms after J point. We then calculated on a 30-minute basis the average ST-height for each lead. Therefore, the ST-height measures are treated as repeated outcome measures, and each individual contributed 48 data points for each of the ten leads. Correlation coefficients [19] for the ST-height measures obtained from ten leads were presented in Table 1. The ST-heights in inferior leads were highly correlated.

**Table 1:** Correlation coefficients for ST-height measurements

	ST-I	ST-II	ST-III	ST-aVF	ST-V1	ST-V2	ST-V3	ST-V4	ST-V5	ST-V6
ST-I	1.00									

ST-II	0.62	1.00								

ST-III	0.21	0.90	1.00							

ST-aVF	0.44	0.98	0.97	1.00						

ST-V1	-0.03	-0.12	-0.13	-0.13	1.00					

ST-V2	0.52	0.35	0.14	0.26	0.68	1.00				

ST-V3	0.49	0.50	0.35	0.45	0.30	0.66	1.00			

ST-V4	0.62	0.70	0.52	0.63	0.19	0.64	0.87	1.00		

ST-V5	0.70	0.81	0.62	0.75	0.04	0.53	0.70	0.91	1.00	

ST-V6	0.69	0.87	0.70	0.82	-0.10	0.39	0.53	0.75	0.92	1.00

### Heart Rate Variability (HRV) Variables

We performed time and frequency domain HRV analysis on the ECG recording, after removing artifacts with standardized visual inspection and statistical filters. We calculated HRV indices from 30-minute segment-specific recordings using the SuperECG package (Mortara Instrument, Inc., Milwaukee, WI) according to current recommendations [20], and used the following HRV indices as measures of cardiac autonomic modulation (CAM): standard deviation of all RR intervals (SDNN, ms), square root of the mean of the sum of the squares of differences between adjacent RR (RMSSD, ms), power in the low frequency range (0.04-0.15 Hz, LF), power in the high frequency range (0.15-0.40 Hz, HF), and the ratio of LF and HF (LF/HF).

### Weather Variables

We obtained individual-level real-time temperature and relative humidity using the HOBO H8 logger (Onset Computer Corporation, Bourne, MA). The real-time temperature and relative humidity were recorded at one-minute intervals initially. For each participant, we calculated 30-minute segment specific averages, corresponding to the PM_2.5 _and Holter measures. Therefore, these weather covariables are treated as repeated measures, and each individual contributed 48 data points for each variable.

### Other Participant-Level Covariables

A standardized questionnaire administered on Day-1 of the study was used to collect the following individual-level information: (a) demographic variables, including age, race, sex, and highest education level; (b) medication uses, including anti-anginal medication, anti-hypertensive medication, and anti-diabetic medication; and (c) physician diagnosed chronic disease history, including CVD (including revascularization procedures and myocardial infarction), hypertension, and diabetes. The averages of the second and third measures of seated systolic and diastolic blood pressures on Day-1 were used to represent a participant's blood pressure levels. Day-1 fasting glucose was measured by Penn State GCRC central laboratory. CVD was defined by anti-anginal medication use or a history of CVD. Hypertension was defined by anti-hypertensive medication use, physician diagnosed hypertension, systolic blood pressure ≥ 140 mmHg, or diastolic blood pressure ≥ 90 mm Hg. Diabetes was defined by anti-diabetic medication use, physician diagnosed diabetes, or fasting glucose ≥ 126 mg/dl. Body mass index (BMI) was defined as the ratio of weight to height squared (BMI, kg/m^2^).

### Statistical Analysis

Two-sample t tests or chi-square tests, as appropriate, were used to compare the distributions of basic demographic variables between participants with and without chronic diseases. We perform a repeated measures analysis using distributed lag models [21-23] under a linear mixed-effects models [24,25] framework, specifying a first-order autoregressive covariance structure to model the correlation between observations from the same participant, to estimate the regression coefficients between 30-minute PM_2.5 _and the ST-height measures. Time was included as a categorical variable to allow flexibility in modeling the relationship between ST-height measures and time. Residual diagnostics were used to assess the appropriateness of modeling assumptions, including normality and homogeneity of variance, and no sizeable departures were detected. In these models, one lag indicates a 30-minute separation between the exposure and outcome. Thus, Lag 0 indicates the spontaneous relationships between PM_2.5 _and the ST-height, and Lag 1 indicates 30 minutes between the PM_2.5 _and ST-height, and so on. We chose a constrained distributed lag model, the polynomial distributed lag model, to reduce the potential collinearity of PM_2.5 _between individual lags using a second-degree polynomial. The linear mixed-effects model framework was chosen because it allows us to explicitly model the expected correlation between measurements taken from the same participant. As the measurements are equally spaced, we used the natural choice for correlation structure, autoregressive order one (AR1), throughout the analyses to account for the potential autocorrelation. A second-degree polynomial for the distributed lag model was used because the amount of variability in the outcomes of interest explained by the air pollution measures is not large, so that the degree of polynomial must be chosen parsimoniously. In our experience, second-degree polynomials perform adequately, which is supported by Schwartz [23]. Another advantage of the distributed lag model is its ability to provide interpretation of the cumulative effects of the lags included in the model, as well as individual lag effects. Because the PM_2.5 _and ECG variables were assessed in parallel over 48 lags (24 hour), we decided a prior to model no more than ten lags, which allowed us to fit the distributed lag model using at least 75% of the data. We started from the largest number of lags (lag 0-10), and reduced the total number of individual lags by back-eliminating the longer lags (e.g., lag 10) one lag at a time until a significant cumulative effect on ST-II was identified (lag 4 in this report). We then identified this model as our final model for all ST-height measurements. All results are expressed per 10 μg/m^3 ^increase in PM_2.5_. In the distributed lag models, basic demographic variables were included as the first step of covariable adjustment. We then included additional adjustment for diabetes, hypertension and CVD. To examine the impact of CAM on PM_2.5 _and ST-height associations, we repeated the models by adjusting for each of the HRV variables. In these models, all time-dependent covariables, such as weather and HRV variables, were entered in the model using the same distributed lag structure as the PM_2.5 _variable. We used SAS statistical version 9 (SAS Institute, Inc., Cary, NC) for all analyses.

## Results

**Figure 1:**
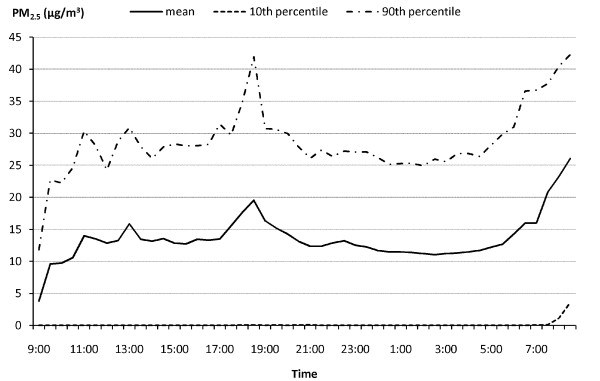
**Time Specific Distribution of PM_2.5 _Exposure over 24 Hours in the APACR Study**.

**Figure 2:**
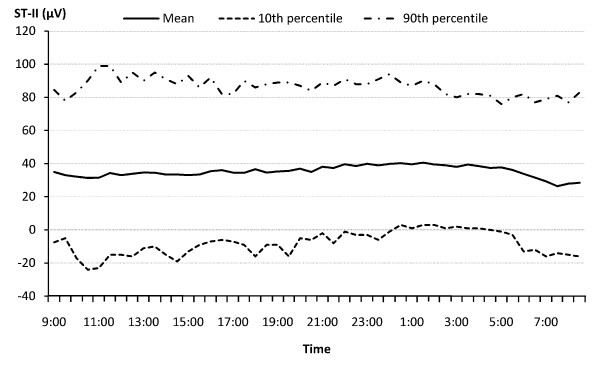
**Time Specific Distribution of ST-II Height over 24 Hours in the APACR Study**.

The demographic and cardiovascular disease risk profiles of the study population are presented in Table 2. The mean age of the participants was 56 years, with 74% non-Hispanic white, 26% minorities (including Blacks, Hispanics, and Chinese), 59% female, and 43% having chronic diseases (primarily hypertension). The prevalence of hypertension, diabetes, and CVD were 34.9%, 7.6%, and 8.5% respectively. At the population level, the distribution of both PM_2.5 _exposure and ST-height measures are approximately normal. The ST-height measures are significantly lower in subjects with chronic disease. Figure 1 and 2 show the time-of-the-day-specific distributions (mean, 10^th ^and 90^th ^percentile) of the PM_2.5 _and ST-height from limb lead II (ST-II), as an example of ST-height measures. Both the PM_2.5 _and ST-height showed sufficient variation within 24 hours, and within time frame between individuals.

**Table 2:** Demographic characteristics and health status of the study population

Characteristic	All subjects	Hypertension, Diabetes, or CVD		
		
		No	Yes	P value
	**N = 106**	**N = 60**	**N = 46**	

Age	56.2 (7.6)	55.6 (8.2)	57.1 (6.8)	0.30

Gender (% Male)	40.6	40.0	41.3	0.89

Race (% White)	73.6	71.7	76.1	0.61

Glucose (mg/dL)	88.8 (25.1)	84.6 (10.0)	94.3 (36.0)	0.09

BMI (kg/m2)	27.7 (5.9)	26.2 (4.3)	29.7 (7.0)	<0.01

CVD (%)	8.5	0.0	19.6	<0.01

Hypertension (%)	34.9	0.0	84.8	<0.01

Diabetes (%)	7.6	0.0	17.4	<0.01

Systolic BP(mm Hg)	121.9 (15.7)	117.1 (11.9)	128.2 (17.9)	<0.01

Diastolic BP (mm Hg)	75.1 (9.2)	73.1 (8.3)	77.6 (9.8)	0.01

College or higher (%)	78.3	73.3	84.8	0.16

Inferior Leads				

ST-II (μV)	35.4 (38.0)	40.7 (38.0)	28.5 (7.0)	<0.01

ST-III (μV)	19.4 (30.6)	22.5 (31.8)	15.4 (28.5)	<0.01

ST-aVF (μV)	27.4 (33.5)	31.6(34.2)	22.0 (31.7)	<0.01

Lateral Leads				

ST-I (μV)	15.5 (17.1)	17.8 (15.3)	12.6 (18.9)	<0.01

ST-V5 (μV)	46.8 (48.6)	55.0 (50.7)	36.1 (43.5)	<0.01

ST-V6 (μV)	28.2 (37.7)	34.4 (38.5)	20.1 (25.3)	<0.01

Septal Leads				

ST-V1 (μV)	40.9 (31.9)	41.4 (33.2)	40.4 (30.1)	0.29

ST-V2 (μV)	90.64 (56.09)	94.3 (56.0)	86.0 (55.9)	<0.01

Anterior Leads				

ST-V3 (μV)	91.8 (65.5)	100.3 (71.3)	80.8 (55.2)	<0.01

ST-V4 (μV)	72.5 (56.8)	81.7 (58.5)	60.7 (52.4)	<0.01

PM_2.5_(μg/m^3^)	13.6 (21.6)	11.9 (14.7)	15.9 (28.0)	<0.01

Temperature (°C)	21.8 (3.5)	21.8 (3.7)	21.7 (3.4)	0.04

Relative humidity (%)	39.7 (12.1)	40.2 (12.3)	39.0 (11.8)	<0.01

Results are presented as mean (SD) for continuous variables and percentage for categorical variables.

**Figure 3:**
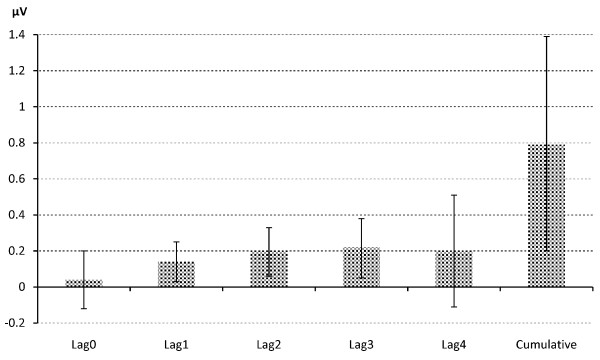
Effect of 10 μg/m^3 ^Increase in Lag 0-4 PM_2.5 _Exposure on ST-II.

The cumulative effect and individual lag effects of PM_2.5 _on each of the ST-height measures are summarized in Table 3 as multivariable-adjusted regression coefficient β (95% CI) associated with a 10 μg/m^3 ^increment of PM_2.5 _exposure. In summary, five leads (ST-I, II, III, aVF, and ST-V6), out of ten ECG leads analyzed, showed a significant positive association between PM_2.5 _exposure and ST-height. Examining the cumulative effect, most of the adverse effect occurred within two hours (lag0-4) after the elevation of PM_2.5_. Specifically, the cumulative effect due to a 10 μg/m^3 ^increase in Lag 0-4 PM_2.5 _concentration on ST-I, II, III, aVF and ST-V6 were 0.29 (0.01-0.56) μV, 0.79 (0.20-1.39) μV, 0.52 (0.01-1.05) μV, 0.65 (0.11-1.19) μV, and 0.58 (0.07-1.09) μV, respectively. To visualize the acute effect of PM_2.5 _exposure on ST-height and its time course, both individual and cumulative effects of lag 0-4 PM on ST-II height are presented in Figure 3, as an example. Based on the cardiac anatomy, we find that all inferior leads (II, III, and aVF) and two out of three lateral leads (I and V6) show significant ST-height elevation. Therefore, our results also indicate that acute PM_2.5 _exposure lead to regional cardiac ischemic changes. Our data also revealed a less consistent individual lag effect. Additional adjustment for chronic disease did not change the pattern of association from the models adjusted only for major demographic and weather-related variables.

**Table 3:** Regression coefficient of ST-height associated with 10 μg/m^3 ^increment of PM_2.5 _concentration.

ST Variable		Lag0	95% CI	Lag1	95% CI	Lag2	95% CI	Lag3	95%CI	Lag4	95% CI	Cumulative	95% CI
Inferior Leads													

ST-II	M1	0.04	(-0.12,0.20)	0.13	(0.02,0.24)	0.19	(0.05,0.33)	0.21	(0.04,0.38)	0.19	(-0.12,0.50)	**0.76**	**(0.17,1.36)**

	M2	0.04	(-0.12,0.20)	0.14	(0.03,0.25)	0.20	(0.06,0.33)	0.22	(0.05, 0.38)	0.20	(-0.11,0.51)	**0.79**	**(0.20,1.39)**

ST-III	M1	0.02	(-0.13,0.16)	0.08	(-0.01,0.18)	0.13	(0.01,0.25)	0.14	(-0.01,0.30)	0.14	(-0.14,0.41)	**0.50**	**(-0.02,1.03)**

	M2	0.02	(-0.12,0.16)	0.09	(-0.01,0.18)	0.13	(0.01,0.25)	0.15	(0.01,0.30)	0.14	(-0.13,0.41)	**0.52**	**(0.01,1.05)**

ST-aVF	M1	0.03	(-0.12,0.18)	0.11	(0.01,0.21)	0.16	(0.03,0.28)	0.17	(0.02,0.33)	0.16	(-0.12,0.44)	**0.63**	**(0.06,1.17)**

	M2	0.03	(-0.11,0.18)	0.11	(0.01,0.21)	0.16	(0.04,0.28)	0.18	(0.03,0.33)	0.17	(-0.11,0.45)	**0.65**	**(0.11,1.19)**

Lateral Leads													

ST-I	M1	0.03	(-0.05,0.10)	0.05	(0.01,0.10)	0.06	(0.01,0.13)	0.07	(-0.01,0.15)	0.06	(-0.08,0.21)	**0.27**	**(-0.01,055)**

	M2	0.03	(-0.05,0.10)	0.05	(0.01,0.10)	0.07	(0.01,0.13)	0.07	(-0.01,0.15)	0.07	(-0.08,0.21)	**0.29**	**(0.01,0.56)**

ST-V5	M1	-0.01	(-0.20,0.17)	0.05	(-0.08,0.17)	0.10	(-0.05,0.26)	0.15	(-0.04,0.34)	0.19	(-0.16,0.54)	**0.48**	**(-0.20,1.15)**

	M2	-0.01	(-0.19,0.18)	0.06	(-0.07,0.18)	0.11	(-0.04,0.27)	0.16	(-0.03,0.35)	0.20	(-0.15,0.55)	**0.52**	**(-0.16,1.20)**

ST-V6	M1	-0.01	(-0.14,0.13)	0.04	(-0.05,0.14)	0.10	(-0.02,0.55)	0.17	(0.02,0.31)	0.25	(-0.02,0.51)	**0.55**	**(0.04,1.06)**

	M2	0.01	(-0.14,0.14)	0.05	(-0.05,0.14)	0.10	(-0.01,0.22)	0.17	(0.03,0.32)	0.25	(-0.01,0.52)	**0.58**	**(0.07,1.09)**

Spetal Leads													

ST-V1	M1	-0.01	(-0.16,0.15)	0.05	(-0.06,0.15)	0.08	(-0.05,0.21)	0.08	(-0.08,0.25)	0.06	(-0.23,0.36)	**0.26**	**(-0.31,0.83)**

	M2	-0.01	(-0.16,0.15)	0.05	(-0.06,0.15)	0.08	(-0.05,0.21)	0.09	(-0.08,0.25)	0.07	(-0.23,0.36)	**0.27**	**(-0.30,0.84)**

ST-V2	M1	-0.07	(-0.31,0.17)	0.11	(-0.04,0.27)	0.22	(0.02,0.42)	0.24	(0.01,0.49)	0.19	(-0.27,0.64)	**0.69**	**(-0.17,1.56)**

	M2	-0.06	(-0.30,0.17)	0.12	(-0.04,0.28)	0.22	(0.02,0.42)	0.25	(0.01,0.50)	0.19	(-0.26,0.65)	**0.72**	**(-0.14,1.59)**

Anterior Leads													

ST-V3	M1	-0.11	(-0.43,0.21)	0.06	(-0.15,0.27)	0.14	(-0.13,0.40)	0.12	(-0.21,0.05)	0.01	(-0.61,0.62)	**0.20**	**(-0.94,1.35)**

	M2	-0.09	(-0.41,0.23)	0.08	(-0.13,0.29)	0.16	(-0.11,0.43)	0.14	(-0.19,0.47)	0.04	(-0.58,0.65)	**0.33**	**(-0.82,1.47)**

ST-V4	M1	-0.09	(-0.27,0.09)	0.03	(-0.09,0.16)	0.11	(-0.04,0.26)	0.14	(-0.05,0.33)	0.13	(-0.21,0.48)	**0.33**	**(-0.34,1.00)**

	M2	-0.08	(-0.26,0.10)	0.04	(-0.09,0.16)	0.12	(-0.04,0.27)	0.15	(-0.04,0.34)	0.14	(-0.20,0.49)	**0.37**	**(-0.30,1.04)**

M1: Adjusted for age, sex, race, temperature, and relative humidity

2: Adjusted for age, sex, race, temperature, and relative humidity, diabetes, hypertension, and CVD

The statistically significant final multivariable-adjusted models (M2, in Table 3) were repeated with additional adjustment for heart rate variability variables, one HRV variable at a time. The HRV adjusted regression coefficients measuring the cumulative effects of PM_2.5 _from 0-4 lags after adjusting for HRV variables are presented in Table 4. In summary, the overall pattern of associations between PM_2.5 _and ST-height did not change substantially with additional adjustment for HRV variables as measures of cardiac autonomic modulation. Therefore, the ST-segment elevation we observed is independent of sympathetic and parasympathetic nervous activity.

**Table 4:** HRV adjusted Lag0-4 cumulative effect of ST-height associated with 10 μg/m^3 ^increment of PM_2.5_.

ST Variable	Regression Coefficients (SE) and 95% CI							
	
	Log-HF	95% CI	Log-LF	95%CI	SDNN	95% CI	RMSSD	95% CI
ST-I	0.31	(0.03,0.58)	0.31	(0.03,0.58)	0.29	(0.02,0.57)	0.31	(0.03,0.58)

ST-II	0.77	(0.24,1.30)	0.77	(0.24,1.30)	0.73	(0.20,1.26)	0.78	(0.25,1.31)

ST-III	0.47	(0.02,0.92)	0.47	(0.02,0.92)	0.45	(0.01,0.90)	0.48	(0.03,0.93)

ST-aVF	0.61	(0.14,1.08)	0.61	(0.14,1.08)	0.58	(0.11,1.05)	0.62	(0.15,1.10)

ST-V6	0.57	(0.07,1.08)	0.57	(0.07,1.08)	0.56	(0.05,1.06)	0.58	(0.07,1.08)

Adjusted for age, sex, race, temperature, relative humidity, diabetes, hypertension, CVD, and each of the HRV indices.

We tested the interaction terms between PM_2.5 _and chronic condition, and none were statistically significant at the 0.05 level (data not shown). Therefore, the effects of PM_2.5 _on ST-height did not differ depending on whether a person had previous health problems. We also performed stratified analysis according to chronic disease status, using M1 in Table 3. We found similar associations by chronic disease status (data not shown). It should be noted that the sample size of this study is small, and individuals with chronic conditions consisted mostly of well-controlled hypertensives. Therefore, the statistical power was limited to detect significant effect modification by chronic disease status.

## Discussion

A large number of epidemiologic studies have found an association between short-term exposure to increased particulate air pollution and CVD morbidity and mortality [1-4,26,27]. However, the mechanisms responsible for such an association have not been fully identified. Previous studies have suggested several promising underlying mechanisms, including cardiac autonomic impairment as measured by lower HRV [13,28-32], ventricular repolarization [5,14,33-37], arrhythmia [38-41], and myocardial ischemia [6-9,42]. The actual time-course from PM exposure to effects on cardiac measures has not been investigated systematically in a community-based sample. Cavallari and coworkers reported an early- and a later-phase HRV response, with the early effects at two hours and delayed effects at nine to thirteen hours after exposure [43]. We reported a sub-acute PM_2.5 _effect on HRV at approximate in four to six hours [13], and an acute PM_2.5 _effect on ventricular repolarization at three to four hours [14]. No studies to date have investigated whether the PM and myocardial ischemia association is mediated through its adverse effects on other ECG parameters, such as CAM.

In this study of community-based individuals, we examined the acute effect and time course of PM_2.5 _on myocardial ischemia. However, we did not limit our examination to the characteristics of clinically manifest ischemia (e.g. ≥ 100 μV for limb leads and ≥ 200 μV for precordial leads of ST-segment elevation), since air pollution could possibly induce ischemic changes to a lesser degree. Our results reveal that PM_2.5 _had a significant positive effect on ST-height from five out of ten leads, supporting that elevated PM_2.5 _levels can lead to higher ST-height in inferior and lateral leads, which can be considered as indications of potential for regional myocardial ischemic damage. These findings are consistent with previous reports [6-9,42], and extend the adverse PM_2.5 _effects from special populations to a community-based sample of healthy individuals, and from ambient-based estimated PM exposures to individual-level measured exposures to PM.

We examined whether the PM and ST-height relationship is chiefly mediated through its adverse effect on CAM. As evidenced by the results presented in Table 4, the PM and ST-height associations in all five leads remained unchanged after adjusting for HRV variables as measures of CAM. Thus, our data did not support that the PM_2.5 _and myocardial ischemia association was majorly mediated through its adverse effects on CAM, at least not from the same lagged effects that we analyzed. However several other mechanisms could explain the adverse effects of PM on myocardial ischemia. First, PM may induce a constriction in the coronary artery. Supporting this hypothesis, Brook and coworkers reported a sudden conduit vasoconstriction after a short-term PM_2.5 _and O_3 _exposure in a sample of healthy volunteers [44]. Mills and coworkers also reported similar patterns supporting the vascular dysfunction effects of PM exposures [45]. Second, PM may also decrease the oxygen-carrying capacity of the blood, as supported by studies reported by several investigators [46-48]. Third, it is also possible that the ST-height changes in response to elevated PM_2.5 _in parallel to its well documented adverse effects on CAM. This is similar to the electrophysiological changes seen in Brugada Syndrome, which is associated with high incidence of sudden cardiac death [49] by means of alternation in repolarizing currents presented on ECG as lower HRV and ST-segment elevation in the right precordial ECG leads V1-V3. Lastly, it is also plausible that PM has a direct adverse effect on cardiac electrophysiological parameters, such as ST-height. More mechanistic studies are needed to investigate the exact pathways from PM exposure to the adverse cardiac electrophysiological effects.

On the time course of PM_2.5 _effects on myocardial ischemia measures, our data consistently indicate that the time course of the adverse effect is within four lags of the exposure window - approximately two hours of elevated PM_2.5 _exposure, similar to that reported by Mills and coworkers [45] on the acute effects of diesel exhaust on vascular dysfunction and impaired endogenous fibrinolysis. Our findings on the time-course of PM_2.5 _effects on ischemia are also consistent with that reported by Cavallari and coworkers [43] on the early effects of PM exposure on HRV. In the same population, time course of PM_2.5 _effects on myocardial ischemia measures are more acute than the effects on HRV [13] and on ventricular repolarization [14]. To our knowledge, this is the first study to demonstrate the time-course of the acute effects of PM_2.5 _on myocardial ischemia in healthy middle aged individuals sampled from communities.

It is worthwhile noting that most of the effect sizes of individual lags within the two hour time frame (lag0-lag4) are smaller than the cumulative effects. As expected, some of the associations between individual lag PM exposure and ST-height are not statistically significant. While individual lag effects may be too small to show an important contribution to the ST-height, the sum of such small effects is large enough to show a significant impact on the ST-height measures. This can be clearly illustrated by Figure 3 as well. On the other hand, the cumulative effect sizes we identified in this study are also relatively small. For example, for every 10 μg/m^3 ^increase in Lag 0-4 PM_2.5 _exposure, the associated increases in ST-II is only 0.79 μV, corresponding to 2% increase in this variable, which has a mean of 35 and SD of 38 μV. Therefore, such small effects on ST-height may not be clinically meaningful. On the other hand, it can be argued that the entire population is exposed to PM_2.5 _on a continuous basis daily. Thus, elevated PM_2.5 _levels can have a huge public health impact. Moreover, the minor effect observed in ST-height in this study was measured in generally healthy individuals. It is possible that PM_2.5 _effect on myocardial ischemia might be greater in individuals with underlying structural heart disease or ischemic heart disease. Future studies should target these clinical subgroups likely to be more susceptible to the effects of PM_2.5_, especially those made more vulnerable by residing near sources of PM_2.5_, e.g. highways.

There are several limitations. First, the APACR study excluded smokers and persons with acute cardiac events within the past six months. Thus, our findings may not apply to smokers or persons with a recent acute cardiac event. Second, the majority of participants reported that they stayed indoors most of the time during the 24-hour study period, except when they had to travel by automobile. This behavior pattern is reflected in the relatively low levels of exposure to PM_2.5_. In general, our participants had limited indoor exposures, such as second-hand smoking. Thus, we were unable to assess whether exposures at much higher levels would exhibit similar associations. However, we purposely used the personal monitors and real-time Holter system to collect the true individual-level exposure and routine ECG data, respectively. We argue that the associations we observed in these individuals are more reflective of their routine exposure and outcome associations. Third, the ECG data from Holter were not collected under a controlled, supine position setting. Thus, the short-term variation of other factors that may impact the ST-height cannot be fully accounted for. However, our study captures the range of activities occurring in real life, including time spent outdoors, time spent commuting in automobile, and various other activities associated with a disease-free community-based individual. Lastly, the pDR estimated PM_2.5 _concentrations over 24 hours were used in this study as an estimation of personal exposures. This nephelometric device responds to the optical properties of the particles it encounters rather than the true particle gravimetric properties. It should be recognized that the optical properties calibrated using Arizona road dust might not be highly representative of the actual PM_2.5 _aerosol the study participants encountered. Previous studies have reported that the Arizona road dust calibrated pDR, identical to the one used in this study, provides a mass concentration estimate in good agreement with that from gravimetric mass-based measures (correlation coefficients > 0.8), but the pDR often yield estimates 10-50% higher than that from a gravimetric mass-based measures[15,16,18]. Based on these validation studies, the personal exposures used in this study might slightly, but systematically, overestimate the true environmental conditions that existed. However, such a systematic overestimation of the true exposures to PM_2.5 _should not have biased the pattern of the observed associations. It can be argued that the reported effect sizes per 10 μg/m^3 ^increase in the pDR estimated PM_2.5 _actually represented effect sizes of only 5.00 - 9.00 μg/m^3 ^increase in the true PM_2.5 _exposure, if pDR systematically overestimates the true exposure by 10-50%, i.e., the reported effect sizes in this study were systematically underestimated.

## Conclusions

In summary, acute exposure to PM_2.5 _at the individual level, is directly associated with higher ST-height measures in inferior and lateral leads, which is indicative of regional ischemic damage potential to the myocardium. Moreover, the time to effects is about two hours after elevated PM_2.5_. The effect of PM on ST-height is independent of major confounding factors and cannot be attributed solely to its effects on HRV as measures of CAM. Overall, these findings support that PM may affect myocardial ischemia, and partly through such a mechanism, PM increases cardiovascular risk, such as sudden cardiac death and myocardial infarction.

## Abbreviations

AR(1): Autoregressive order one; BMI: Body Mass Index; CAM: Cardiac Autonomic Modulation; CVD: Cardiovascular Diseases; ECG: Electrocardiography; GCRC: General Clinical Research Center; HF: Power in the High-Frequency range (0.15-0.40 Hz); HRV: Heart Rate Variability; LF: Power in the Low-Frequency range (0.04-0.15 Hz); PM: Particulate Matter; RMSSD: Square Root of the Mean of the Sum of the Squares of Differences between adjacent RR intervals; SD: Standard Deviation; SDNN: Standard Deviation of Normal-to-Normal RR intervals.

## Competing interests

The authors declare that they have no competing interests.

## Authors' contributions

FH performed statistical analysis and drafted the manuscript. MLS built the statistical models and wrote the statistical methods section. RS contributed to the data collection and revision of the manuscript. EOB and ANV suggested major revisions of the manuscript. RWW contributed to the air pollution data collection and revision of the paper. RW contributed to the interpretation and methods of the statistical modeling. WEC contributed in the revision of the paper and interpretation of the ECG variables. DL, principle investigator of the APACR study, conceptualized the hypothesis and conducted the data collection, made major contributions in the interpretation of the results, and writing of the paper. All authors read and approved the final manuscript.
